# Non-Hodgkin’s Lymphoma Presenting as Esophageal Stenosis in a Pediatric Patient

**DOI:** 10.1097/PG9.0000000000000213

**Published:** 2022-07-25

**Authors:** Jeffrey Lee, Hamza H. Khan, Morgan McBee, Nagraj Kasi

**Affiliations:** From the Division of Pediatric Gastroenterology, Hepatology, and Nutrition MUSC Charleston, South Carolina Department of Radiology and Radiological Sciences

**FIGURE 1.**
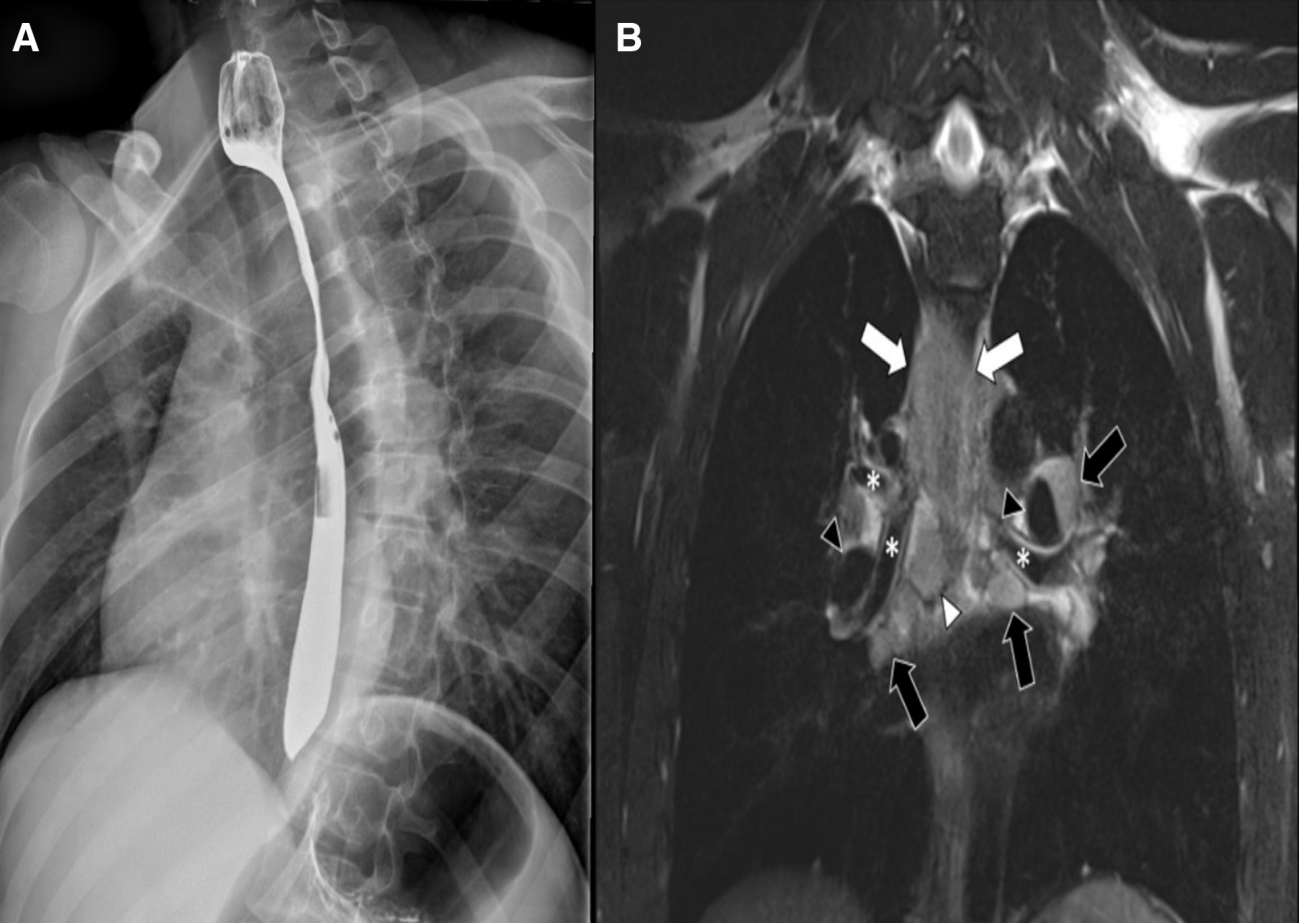
A) Esophagram performed at an outside hospital revealing a 7-cm stricture beginning at the thoracic outlet and extending caudally. B) Coronal MR image (T2-weighted HASTE) again demonstrates the circumferential esophageal wall thickening (white arrows). There are enlarged hilar lymph nodes bilaterally (black arrows) along the bronchi (white asterisks) and pulmonary arteries (black arrowheads) as well as an enlarged subcarinal lymph node (white arrowhead). Given the presence of lymphadenopathy outside the drainage pattern of the esophagus, lymphoma is the leading differential consideration on imaging with primary esophageal malignancy favored to be less likely.

**FIGURE 2.**
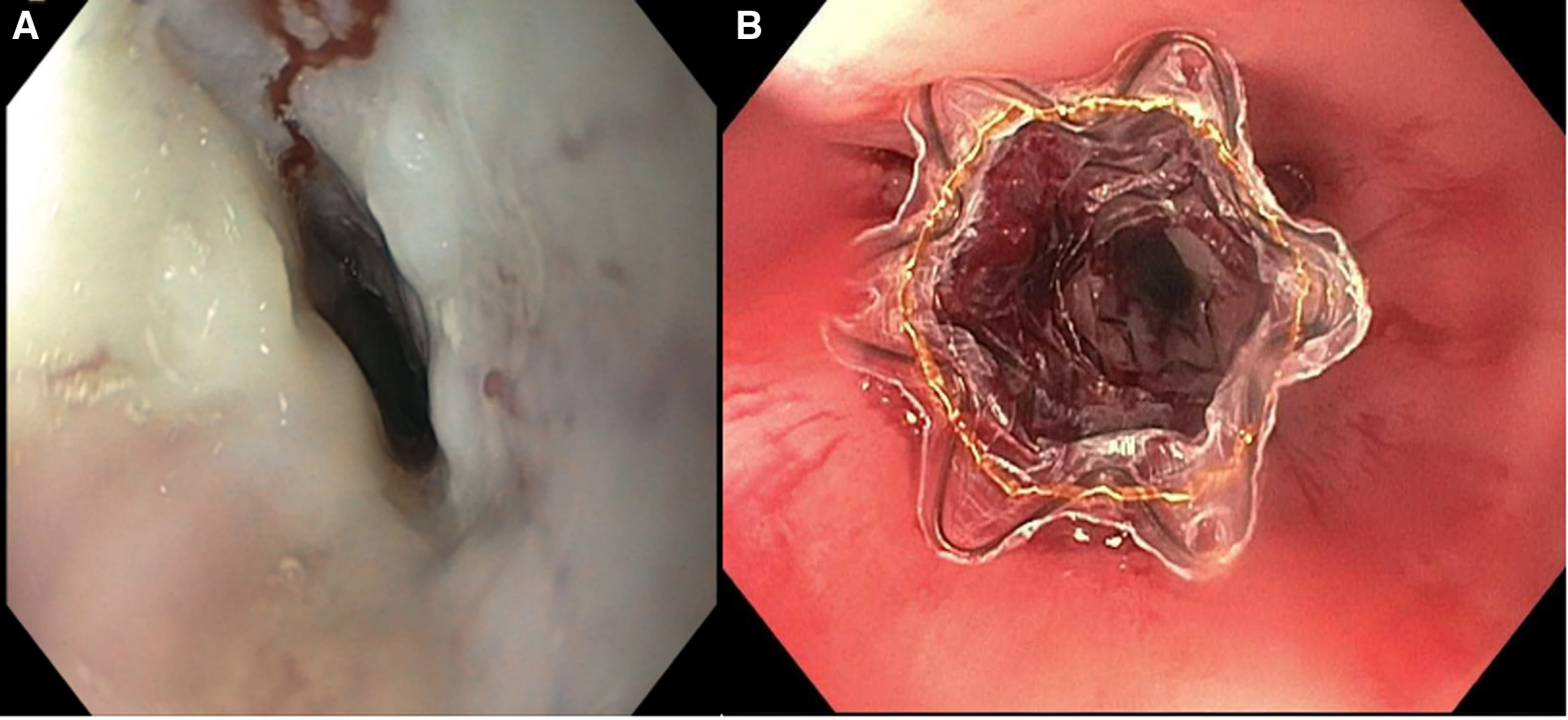
A) Esophageal stricture pre-stent placement. B) Esophageal stricture post-stent placement.

A 14-year-old previously healthy male presented with acute onset of esophageal food impaction. Patient endorsed progressively worsening dysphagia for 4 weeks, initially with solids but now with liquids. He denied any other symptoms including fevers or weight loss and had no previous history of allergies, eczema, or asthma. An esophagram at an outside hospital revealed a 7-cm narrowing (Fig. 1A), and he was referred to pediatric gastroenterology. However, due to worsening symptoms, he presented to the emergency department. Upper endoscopy revealed a proximal esophageal stricture (Fig. 2A) that could not be traversed by a standard-sized endoscope. A neonatal endoscope successfully passed a measured 7-cm stricture. Endoscopic evaluation of mucosa proximal and distal to the stricture was unremarkable, and biopsies were normal. Because of the short duration of symptoms with a long stricture, lack of endoscopically visualized inflammation, and normal biopsies, a chest radiograph was performed and revealed possible mediastinal widening. Magnetic resonance imaging of the chest confirmed circumferential esophageal wall thickening of the upper thoracic esophagus involving up to 8 cm in length and enlarged surrounding lymph nodes suspicious for lymphoma versus primary esophageal malignancy (Fig. 1B). Computed tomography of the chest confirmed these findings. Endoscopic ultrasound with fine needle aspiration of one of the lymph nodes was performed and an esophageal stent was placed (Fig. 2B). Biopsies revealed atypical B cells consistent with malignant lymphoma. He was ultimately diagnosed with non-Hodgkin’s lymphoma and is doing well following initiation of chemotherapy.

Primary involvement of the esophagus is a rare manifestation of lymphoma, with few published cases in adults and even fewer in children (1). Less than 1% of gastrointestinal lymphoma cases involve the esophagus, and of these, only 0.2% of patients present with primary esophageal presentation (2). Given its rarity, pediatric incidence rates are unknown. Presenting symptoms include dysphagia, abdominal pain, and weight loss (3–5). Reported complications of associated strictures include perforation and tracheaesophageal fistula (6,7). In our case, the patient had no classic symptoms of lymphoma; however, due to the lack of risk factors associated with eosinophilic esophagitis (EOE), lack of endoscopic/histologic evidence of EOE, and short duration of symptoms with a lengthy stricture, we suspected another more insidious etiology prompting further imaging studies. Careful history can help distinguish unusual diagnoses and expedite necessary imaging and treatment as in this case.
